# Supplementary material for: Nodding syndrome, a case-control study in Mahenge, Tanzania: Onchocerca volvulus and not Mansonella perstans as a risk factor
Source: PLoS Negl Trop Dis. 2023 Jun 20;17(6):e0011434. doi: 10.1371/journal.pntd.0011434 (PMC10313037; doi:10.1371/journal.pntd.0011434)
Supplement: S1 Table — (DOCX) [file pntd.0011434.s001.docx]

# Supplement 1. Matching of cases with controls of neighbouring villages.

Most cases (80.5%) were age- and sex-matched to controls from the same village (Table 1). However, some cases were matched to controls from neighbouring villages due to a lack of controls available in their villages, particularly in Mzelezi (10/15) and Isyaga (5/13) villages.

**S1 Table . Cases and controls per village.**

| **Village** | **Cases** | **Controls** | **Matching outside the village** |
| --- | --- | --- | --- |
| **Ebuyu** | 10 | 25 | One case matched with a control from Isongo. |
| **Euga** | 0 | 1 |  |
| **Isongo** | 28 | 31 |  |
| **Isyaga** | 13 | 8 | Five cases matched with controls from Mgolo (2), Ebuyu (2) or Isongo (1). |
| **Mdindo** | 9 | 10 | Two cases matched with controls from Ebuyu or Isongo. |
| **Mgolo** | 12 | 14 |  |
| **Msogezi** | 13 | 15 | Two cases matched with controls from Mdindo or Euga. |
| **Mzelezi** | 15 | 7 | Ten cases matched with controls from Ebuyu (6), Euga (3) or Sali (1). |
| **Sali** | 6 | 13 | One case matched with a control from Isongo. |
| **Vigoi** | 7 | 8 | One case matched with a control from Ebuyu. |
| **Total** | 113 | 132 | 22/113 (19.5%) cases matched with controls from neighbouring villages. |

Case – Person with epilepsy; Control – Person without epilepsy.
