# Supplementary material for: Nodding syndrome, a case-control study in Mahenge, Tanzania: Onchocerca volvulus and not Mansonella perstans as a risk factor
Source: PLoS Negl Trop Dis. 2023 Jun 20;17(6):e0011434. doi: 10.1371/journal.pntd.0011434 (PMC10313037; doi:10.1371/journal.pntd.0011434)
Supplement: Attachment — Submitted filename: 11.04.23response to reviewersFinal.docx [file pntd.0011434.s003.docx]

**Response to Reviewers**

**Reviewer #1**

Objectives of the study is clearly articulated with a testable hypothesis.

1) The authors report that informed consent was obtained, however detail report is missing. Please specify (preferably under a separate sub-heading) the details of IRB (name and #) that approved the study.

**Response:**

**Thank you for pointing this out. We now included the following ethical statement:**

# **Ethical Approval Statement**

**The study was conducted according to the guidelines of the Declaration of Helsinki. Ethical approval was obtained from the Ethics Committee of the National Institute for Medical Research, Tanzania (NIMR/HQ/R.8a/Vol.IX/3746) and the Ethics Committee of the Antwerp University Hospital, Belgium B300201837863.**

2) In the Discussion line 194-195: “…Ov16 seropositive persons were also more likely to have a nuclear family member with epilepsy, suggesting that the association is likely related to onchocerciasis exposure.” Furthermore, the authors note that genetics might not play an important role in clustering of epilepsy. Among cases, the percentage of individuals with family history of epilepsy in another immediate family member and nuclear family member is 18.9% and 17.7% respectively, where as among in-Law with epilepsy is 7.1%. Thus, overall incidence of epilepsy is higher among related (1st and 2 nd degree relatives) family members than unrelated.

Thus, the clustering of epilepsy in family could be due to a genetic predisposition and not due to common parasitic exposure. Another possibility is that there could be increased genetic risk within families for Onchocerca parasite infection. The study did not evaluate this relationship and thus, authors should consider modifying their statement.

**Response**

**Ov16 seropositive persons were more likely to have a nuclear family member with epilepsy, which suggests that the association is likely related to onchocerciasis exposure. Therefore, we propose to keep this sentence. Moreover, the association between epilepsy and a family member with epilepsy disappeared once an interaction term with Ov16 seropositivity was added to the model. We adapted the discussion:**

**“*The length of residence in the village was also correlated with the Ov16 seroprevalence (cor = 0.30, p < 0.0001). The latter was also correlated to a family history of seizures (cor = 0.32, p < 0.0001). When added to a univariable logistic regression adjusted for age, sex and village type, having a first-degree relative with epilepsy was strongly associated with Ov16 positivity (adjusted OR: 15.02, 95% CI: 2.97-274.7; p = 0.0094) but not significantly related to having a second-degree relative (p = 0.21) or an in-law with epilepsy (p = 0.25). Similarly, a family history of seizures lost all significance when an interaction effect with Ov16 positivity was added to the conditional logistic regression.*”**

**We agree with the reviewer that the terminology “nuclear family member” can be clarified as “first-degree” and “second-degree” relatives. We adapted the paper accordingly.**

**Ultimately, the higher epilepsy prevalence among 1st and 2nd-degree relatives could suggest that the clustering of epilepsy in families could be due to a genetic predisposition. Hence, we now state in the discussion:**

**“*The higher epilepsy prevalence among relatives of cases could also indicate that the clustering of epilepsy in families could be due to a genetic predisposition, with a possible increased genetic risk for an Onchocerca parasite infection within certain families. Still, the association between epilepsy and having a family member with epilepsy was lost once the conditional regression was adjusted for Ov16 seropositivity*.”**

3) In Discussion, line 237-245, the authors suggests reverse causation as a reason for the association between M.perstans and nodding syndrome in South Sudan. Similar argument could be made for increase prevalence of O.volvulus among individuals with NS. In this case-control study, it cannot be ruled out if the association between Onchocerca v. and epilepsy is due to increased risk of infection in persons with epilepsy. One could consider a scenario where persons with epilepsy have an increased functional disabilities and immobility and thus, are more likely to be bitten by vector and at increased risk of OV infection. Request authors to consider such a reverse causation and discuss.

**Response**

**Reverse causation is extremely unlikely to explain the relationship between onchocerciasis and epilepsy. Indeed, two retrospective studies in Cameroon showed a temporal and dose-response relationship between a young child’s *O. volvulus* microfilarial load and the development of epilepsy later in life. This relationship was not found with *M. perstans.* Moreover, persons with epilepsy tend to stay away from rivers because of the risk of drawing and, therefore, less exposed to blackfly bites. There could be some persons with epilepsy who wouldn’t be able to move away from the flies when they are having certain types of seizures, potentially increasing their chance of being bitten. However, we believe this happens to a small extent.**

***See references 47 and 59.***

4) M. perstans infection was assessed by examining dried blood for microfilariae under a microscope. Need to clarify the use of anti-parasitic diethylcarbamazine and albendazole or doxycycline. Prior treatment could affect the presence of M. perstans and lower the risk of active infection. Please discuss if prior treatment of M. perstans was assessed.

**Response**

**We now state in the discussion:**

**“*Diethylcarbamazine and doxycycline were never distributed in Mahenge. As lymphatic filariasis is not endemic in the area, mebendazole is not given to adults; nevertheless, it is possible that preschool- to school-aged children receive the former drug or albendazole once or twice a year as part of the deworming programme (47, 48). Aside from the fact that our study population were adults (median: 28.3, IQR: 22.0-34.0), this treatment is unlikely to have had a substantial impact on M. perstans prevalence. Albendazole has been shown to exert little to no effect on a M. perstans infection, even in combination with ivermectin (17), and mebendazole should be given daily for several weeks to exert an effect (17, 46).”***

5) The conclusions are in line with the results except for the semantic problem mentioned above. The limitations of the study are well established and relevant. The authors’ discussion is well conducted. Although it seems clear that they have a strong view on the issue, they have good arguments and reasoning to try to explain their results. Moreover, this well-conducted discussion is totally focused on public health and will certainly lead to further research and public health actions on this topic.

**Response**

**Thank you!**

**Reviewer #2**

I have read with interest your manuscript title: Nodding Syndrome, a case-control study in Mahenge, Tanzania: Onchocerca volvulus and not Mansonella perstans as a risk factor.

I appreciate your efforts towards understanding nodding syndrome, a disorder that has devastated poor communities in northern Uganda, and South Sudan in recent times.

I have a few suggestions that may help improve further the quality of the manuscript: I will refer to Onchocerca volvulus as OV, and Mansonella perstans as MP.

1. The title of the study seems to suggest the putative risk factors are “either OV or MP” and nothing else! But it may be that of all risk factors, this study investigated OV and MP and of the two, OV is the risk factor. Be that as it may, the title may not have to suggest the central theme of the findings. You may want to review the title to minimise any form of an “either or” fallacy.

**Response**

**Using “either or” in the title could lead to confusion, as persons only looking at the title of the article could get the impression that the association between onchocerciasis and epilepsy is still not established. Our title states that OV is “a risk factor”, not “the risk factor”. Therefore, we would like to keep the title as it is.**

2. In the methods section – both abstract and main text, you refer to having conducted logistic regression analyses, and you report that you adjusted for sex, age and residence, and yet these are also the variables you matched on during recruitment. You may want to explain further why you did not use “conditional logistic regression” but ordinary logistic regression and why you are adjusting or controlling for the same variables you already match on! Refer to line 137 for example. Second, you may want to explain why you matched on these variables during recruitment, and what was the ratio of the matching? 1:1 or 1:2 etc. and the reasons you chose that ratio for your match.

**Response**

**We agree with the reviewer that a conditional logistic regression could be more relevant and adapted the paper accordingly. The results significance remained unchanged, as shown in Table 3.**

**Regarding the matching, we now state:**

***“Cases of epilepsy and controls without the condition were well-matched for sex (p = 0.97), age (p = 0.64), village type (1.00) and ethnicity (p = 0.86). Nevertheless, no controls were available to be matched by age and sex for 22 cases within certain villages, particularly Mzelezi (10) and Isyaga (5) villages. Hence, these cases were matched with controls from neighbouring villages (Supplement 1). The search for persons without epilepsy led to the recruitment of slightly more controls (132) than cases (113), with 19 cases (16.8%) matched to two controls.”***

3. The results may be totally different, the same or similar when a different analytical approaches are used – conditional versus ordinary logistic regressions. I therefore think that further review of the results and discussions would be appropriate after the analytical approaches have been clarified on. But just briefly, you may want to review and report mean with standard deviations and median with interquartile ranges. Note that in line 136 mean is reported with IQR.

**We agree with the reviewer and now implement a conditional logistic regression. We thank the reviewer for noticing the typo on line 136. It has been corrected.**

**Reviewer #3**

The objectives of this study are clearly stated even if there is a slight problem of semantics. Indeed, the authors sometimes speak of a “causal” role when it is an observational study. They need to change some sentences and be careful about the conclusions drawn. The design of the study is adapted to the objective. The population is well described and the sample size seems correct. However, the authors should indicate how and why this size was chosen.

The statistical analyses are also adequate for the purpose, but the authors should be more specific in their method. In particular, the different models used and the variables included in these models.

Ethical and regulatory requirements are well respected.

**Response**

**Thank you for the review. We clarified our methodology and statistical analysis and now implement a conditional logistic regression (matched for age, sex and village) and a sensitivity analysis.**

The analyses presented correspond well to the analysis plan in the method. In general, the results are simple and well presented. Some of the results are presented in easy-to-read tables except for Table 3, which does not clearly indicate the models used. An improvement in the presentation of the method would improve the understanding of some results.

Moreover, no figures are necessary for a good understanding of the results.

**Response**

**Thank you. We now clearly state the model used on the methodology and Table 3.**

COMMENTS

General comment

The authors of the paper “Nodding Syndrome, a case-control study in Mahenge, Tanzania: Onchocerca volvulus and not Mansonella perstans as a risk factor” conducted a study to investigate whether a parasite (Mansonella perstans) previously associated with NS in Sudan was also associated with NS in Tanzania. In view of the data known to date on the subject, all explorations with an etiological aim on NS are of great scientific interest given the current level of our knowledge on this subject. The authors are therefore to be congratulated on the completion of this work Overall the work is well written and the method seems to have been good even if on reading it some details are missing to improve the understanding of the paper. In addition, some of the conclusions drawn do not always seem to be in line with the work carried out. Please pay attention to this. I will start with general comments on the work and then move on to more specific comments in the manuscript.

From my perspective, there is a small discrepancy between the title (which refers to both Onchocerca volvulus (OV) and Mansonella perstans (M.perstans)) and the objective (which seems to focus on M.perstans). Please modify either one and keep it in mind throughout the work, especially in the conclusions you draw.

**Response**

**The objective of the study was indeed to determine the potential role of *M. perstans* as a potential risk factor for nodding syndrome and confirm *O. volvulus* exposure as a risk factor.**

Throughout the work, (introduction and conclusion of the abstract; introduction of the work etc.) you say that you were trying to determine whether the M.perstans could play the sole causal role in the development of NS. How could you prove this with this type of epidemiological study? I would suggest that you were looking to see if it could be a factor associated with the occurrence of NS. Indeed, the term “causal” cannot be used in an observational survey.

**Response**

**We agree that we cannot use the term “causal”. We now state in the introduction:**

***“we investigated whether an infection with M. perstans could be a risk factor in the development of NS in villages affected by NS in Mahenge, Tanzania”***

**And in the discussion:**

***“The first step to understanding the pathogenesis of NS is to identify a common risk factor at all sites where this condition is reported. M. perstans is likely not endemic in Mahenge and, therefore, cannot play a co-factor role in developing NS in the area. Therefore, based on our findings in Mahenge, M. perstans is unlikely to be a potential main and sole cause of NS. However, our study cannot exclude that M. perstans could be a co-risk factor for NS in an M. perstans endemic area. Evidently, more research about M. perstans is needed to determine its ecology, pathogenicity, and link with other infections, including onchocerciasis.”***

**And in the abstract: “In contrast to *O. volvulus*, *M. perstans* is most likely not endemic to Mahenge and, therefore, cannot be a co-factor for NS in the area. Hence, this filaria is unlikely to be the primary and sole causal factor in the development of NS. The main risk factor for NS remains onchocerciasis.”**

In the section method you say that the controls were matched to the cases on some criteria but can you tell us more? Was it a one-case, one-control match? If so, why were there 113 cases for 132 controls? How was the number of subjects needed for the Mansonella perstans study calculated?

In general, it would be important to give a little more detail in the method and to be precise. This problem also causes problems in understanding the results.

**Response**

**The plan was to match cases and controls by age and village but because of COVID-19 complot theories circulating in the community it was difficult in certain villages to always find a control for every case. Hence, some cases were matched with controls from neighbouring villages. We now state in the methodology:**

***“Persons with epilepsy, including NS and Nakalanga Syndrome, were individually matched with controls by age (± three years), sex and village (if possible, from the same neighbourhood). When a matching control was not available in the village of a case, one was sought in adjacent villages.”* And *“Some epilepsy cases were matched with controls from neighbouring villages, as no controls were available within the same village. Hence, a sensitivity analysis of the village matching was performed by adjusting the regression for the village of cases and controls.”***

**And in the results:**

**“*Cases of epilepsy and controls without the condition were well-matched for sex (p = 0.97), age (p = 0.64), village type (1.00) and ethnicity (p = 0.86). Nevertheless, no controls were available to be matched by age and sex for 22 cases within certain villages, particularly Mzelezi (10) and Isyaga (5) villages. Hence, these cases were matched with controls from neighbouring villages (Supplement 1). The search for persons without epilepsy led to the recruitment of slightly more controls (132) than cases (113), with 19 cases (16.8%) matched to two controls*.” And “*The sensitivity analysis performed for the village matching did not impact the significance of any findings*.”**

**And in the discussion:**

**“*The initial plan was to match cases and controls 1:1. However, because of COVID-19 complot theories circulating in the community at the time of the study, it was difficult in certain villages to find village-matched controls for every case. We, therefore, added controls from neighbouring villages*”.**

Below are some minor revisions in the form of specific comments.

Specific comments

Comment 1

In the introduction section, line 47, you state in the text NS for “nodding seizures” and later you use it to refer to “nodding syndrome”. Do you consider a syndrome and a seizure to be the same? Or is this a mistake? If so, please correct.

**Response**

**Nodding seizures is the type of seizures. To meet the definition of nodding syndrome the person needs to present nodding seizures but also meet the remaining criteria of nodding syndrome. We clarified this in the text.**

Comment 2

Line 120 (methods): Can you indicate the sensitivity and specificity of the test used for M.perstans at this level? Even if you want to say it again in the discussion.

**Response**

**The sensitivity of a smear exam for the diagnosis of *M. perstans* infection is about 78 %. The specificity is 100% if the smears are read by a well-trained laboratory technician.**

**We now included in the methods: “*The sensitivity of a microscopic examination of a blood smear to detect a M. perstans infection is estimated to be 78% (32), while the specificity is close to 100% when a well-trained laboratory technician performs the test*”.**

Comment 3

Can you elaborate on the method? Did you run several models (one model for each variable, adjusted for age, sex and type of village) or were all variables included in the same model? Also, in the results, you give an OR for all epilepsies and an OR for NS only. Is it the same model? The same adjustment variables?

**Response**

**Thank you for the useful comment. We now clarify the methodology:**

***“A conditional logistic regression model incorporating the case-control matching factors (sex, age and village) for epilepsy status was run for each clinical characteristic of cases and controls, M. perstans infection, O. volvulus exposure and relevant sociodemographic variables. Odds ratios (OR), 95% confidence intervals (95% CI) and p-values were presented. Some epilepsy cases were matched with controls from neighbouring villages, as no controls were available within the same village. Hence, a sensitivity analysis of the village matching was performed by adjusting the regression for the village of cases and controls. Variables with < 5% frequencies were compared using Fisher's exact test instead of the regression and p-values presented.***

***The conditional model and Fisher's exact test were implemented a second and a third time for probable NS and for OAE by excluding from the analysis the persons with epilepsy that did not meet the criteria of these definitions. The controls of the persons with epilepsy excluded from the NS or OAE models were individually matched to the closest persons with epilepsy meeting the NS or OAE criteria, respectively.”***

In lines 128 and 129 you mention M.perstans among the variables included in the regression model yet you have no cases of M.perstans! Can you be more precise? In other words, why include it in the models?

**Response**

**We now clarify in Table 3 that we left out *M. perstans* as a variable in the model.**

When you talk about exposure to onchocerciasis, are you referring to OV16 or the skin manifestations of the disease? Be specific each time.

**Response**

**When we mention exposure to onchocerciasis, we mean exposure to *O. volvulus*-infected blackflies. Kindly find in the methodology:**

***“Samples were collected, air-dried and stored at 4ºC until handled for further analysis to identify M. perstans infection and O. volvulus exposure. The latter involved the detection of IgG4 antibodies recognising O. volvulus Ov16 antigen in a dried blood spot by enzyme-linked immunosorbent assay (ELISA), using the commercially available Ov16 IgG4 ELISA kits (Standard Diagnostics Gyeonggi-do, Republic of Korea) and following its manufacturer instructions.”***

The answer to these questions may be in Table 3, which assumes that each variable has been adjusted only for age, sex and village. Is this the case? **Yes, indeed**

If yes, why did you not make a model taking into account all relevant variables? **To prevent overfitting the model, and also because some variables had missing values (stated in Table 3), which would have decreased considerably the sample size. For instance, a few cases (9) and controls (12) were not tested for Ov16 antibodies.**

If not, what exactly are the variables included in the model(s)?

In any case, please put this information more clearly in the method and if possible present the results of the more complete models. **Thank you for the helpful review, we clarified the methodology accordingly.**

Comment 4

In discussion: line 195, you explain that the fact that they are neighbours of the cases could explain why a considerable proportion of the in-laws have epilepsy. Of the in-laws with epilepsy in your sample, what percentage live in the neighbourhood of their NS case?

**Response**

**Indeed in-laws often live in the neighbourhood of the NS case. This means they also are more exposed to *O. volvulus-*infected blackflies and therefore, they are more at risk for developing epilepsy. However, we do not know the exact percentage of them that live in the neighbourhood of the NS case. Moreover, we believe that persons with epilepsy are more likely to have in-laws with epilepsy due to epilepsy stigma. Healthy families avoid marrying persons with epilepsy in Mahenge, and siblings of persons with epilepsy may be left with no options but to marry a person with epilepsy. An ongoing qualitative study on epilepsy and onchocerciasis in the area may provide more insightful information on the topic.**

Comment 5

Discussion: Line 246 to 249. You mention poverty as a possible explanation for the link that has been found between M. perstans and NS in Sudan. Is your study population not also poor? And in other places where NS is found? So I don’t understand your argument.

**Response**

**Indeed, all populations where NS is occurring are poor. Therefore, poverty is certainly not the only reason explaining the link between NS and *M. perstans* in South Sudan. For example, parasitic tolerance induced by *O. volvulus* infection could play a role.**

**We now state, “*Factors associated with poverty also potentially played a role in the association between M. perstans infection and NS*”.**

**Reviewer #4**

Abstract

-	Lines 13-14: I suggest you change epilepsy with onchocerciasis-associated epilepsy (OAE) since OAE includes epilepsy, NS and Nakalanga syndrome. Here, your statement seems like epilepsy is synonymous to NS. Your new statement should read “Cases of onchocerciasis-associated epilepsy (OAE) were identified in villages affected by NS in Mahenge, Tanzania, and matched with 14 controls without OAE of the same sex, age and neighbourhood.”

**Response**

**We prefer to keep the text as it is, as a person with epilepsy must meet the OAE criteria to have OAE. Previous surveys in the Mahenge area have shown that most persons with epilepsy meet the OAE criteria but not all. We also present how many people have OAE in our study now:**

***“Forty-three (38.1%) epilepsy cases met the criteria of probable NS, and 106 (93.8%) had OAE (Table 2).”***

**We followed the reviewer's advice in analysing OAE, besides overall epilepsy and probable NS.**

-	Line 19-20: Similar to the comment above change epilepsy with OAE. Also, you meant were men and women, respectively instead of were men, respectively; please correct. Your new statement should be “A total of 113 OAE cases and 132 controls were enrolled, of which 56 (49.6%) and 64 (48.5%) were men women, respectively”.

-	Line 20: Please add “respectively” after controls.

**Response**

**We indeed mean men, as we mention how many of the cases and control were men. To clarify, we changed the order of the sentence to: “*A total of 113 epilepsy cases and 132 controls were enrolled, of which, respectively, 56 (49.6%) and 64 (48.5%) were men*.”**

-	Line 26: Please remove the word “primary” since there is no definitive evidence that OV is the primary casual factor for NS; only association exists.

-	Line 26-27: Please remove the statement “The primary risk factor for NS remains onchocerciasis”. See the previous comment.

- **Response**

**We are stating that *M. perstans* cannot be the primary and sole causal factor. We are nor referring to OV. We agree that we are only studying the association of *M. perstans* and *O. volvulus* to epilepsy in Mahenge, therefore, we adjusted the abstract accordingly:**

**“*We aimed to determine whether the latter parasite could be associated with the NS epidemic in Mahenge*.” And “*In contrast to O. volvulus, M. perstans is most likely not endemic to Mahenge and, therefore, cannot be a co-factor for NS in the area. Hence, this filaria is unlikely to be the primary and sole causal factor in the development of NS. The main risk factor for NS remains onchocerciasis*.”**

Lines 29-36: Please change “epilepsy” with “OAE”.

**Response**

**See the response above; we propose to keep epilepsy while adding analysis for OAE.**

-	Line 37: This statement is too strong “In conclusion, onchocerciasis remains the most likely cause of NS”. Please consider to revise.

**Response**

**We agree. We now state, “In conclusion, onchocerciasis remains the main risk factor for NS”.**

Introduction

-	Lines 30-40: Please add “s” to 1960. It should be in “1960s”.

**Response**

**Thank you for noticing. Changed to 1960s.**

-	Lines 46-48: You should either describe the complete features of the OAE (epilepsy, NS and Nakalanga syndrome) or just mention that OAE include epilepsy, NS and Nakalanga syndrome. Stunted growth and delayed puberty are not the only features of Nakalanga syndrome that makes it distinct, NS also presents with similar features.

**Response**

**We now state “*OAE presents a broad spectrum of clinical manifestations, including NS and Nakalanga syndrome (9*)”.**

Methods

-	Lines 91-92: Please revise the statement “Epilepsy cases and controls without epilepsy were people who screened positive or negative for epilepsy in the previous door-to-door surveys, respectively” to “OAE cases and controls were people who screened positive or negative for OAE in the previous door-to-door surveys, respectively”

**Response**

**See above, we prefer to keep the term epilepsy as not all cases of epilepsy may have met the OAE criteria**

-	Lines 101-103: Please add “thoracic dysmorphia” and “endocrine dysfunction” to the features of Nakalanga syndrome.

**Response**

**We now state: “ A case of Nakalanga Syndrome was defined as a person with a combination of stunted growth, delayed or absent external signs of secondary sexual development (endocrine dysfunction) and mental impairment, often associated with epileptic seizures and facial and thoracic dysmorphia (28).”**

Results

-	Please change “epilepsy” with “OAE” throughout where necessary. See previous comments.

**Response**

**We prefer to keep the term epilepsy while adding an additional analysis for OAE.**

Discussion

-	Line 183: “1960s”.

**Response**

**Changed to 1960s.**

-	Line 185: change “epilepsy” with “OAE”. Also, for consistency, present cases before controls.

-	Please change “epilepsy” with “OAE” throughout where necessary.

**Response**

**Adapted accordingly.**

-	Line 207: Please change “palpitation” with “palpation”.

**Response**

**Thank you for noticing. Changed to palpation.**

Conclusion

Lines 286-287: Please change “the most likely” to “a possible”.

**Response**

**We now state: *“Onchocerciasis remains the main risk factor for NS and other forms of onchocerciasis-associated epilepsy”.***

The study is of importance for the scientific community and the paper is well written. If all comments addressed by the authors, the paper is suitable for publication.

**Response**

**Thank you for your review. We believe we responded appropriately to all the comments of the reviewers.**
