# Supplementary material for: Nodding syndrome, a case-control study in Mahenge, Tanzania: Onchocerca volvulus and not Mansonella perstans as a risk factor
Source: PLoS Negl Trop Dis. 2023 Jun 20;17(6):e0011434. doi: 10.1371/journal.pntd.0011434 (PMC10313037; doi:10.1371/journal.pntd.0011434)
Supplement: Attachment — Submitted filename: 24.05.23ResponsReviewers Mansonela.docx [file pntd.0011434.s004.docx]

**Reviewer 1**

No comments

**Reviewer 2**

**Reviewer**

Are the objectives of the study clearly articulated with a clear testable hypothesis stated? The objectives are clearly stated and testable.

Is the study design appropriate to address the stated objectives? Yes, it is appropriate.

Is the population clearly described and appropriate for the hypothesis being tested? Yes, the population has been clearly described.

1. Is the sample size sufficient to ensure adequate power to address the hypothesis being tested? I have not seen efforts towards stating what the power of the study would be to identify a statistically significant difference between variables being tested by cases - control, status. Some statement of power and sample size calculation will help readers appreciate the study findings better. I find aspects of matching ratio in the response to reviewers comments but can hardly find these in the main manuscript where they should actually be well embedded as the manuscript is what the readers will interface with once the manuscript is published.

**Response**:

We now included a statement about sample size.

We added to the methodology:

*“In Mundri County, South Sudan, a significant association was found between NS and an M. perstans infection, with an odds ratio of 3.2 (p = 0.005). Among the NS cases, 52% (36/69) were infected with M. perstans, compared to 31% (20/65) controls without epilepsy (13). Assuming a similar prevalence in Mahenge, at least half (50%) of NS cases and one-fourth (25%) of controls are expected to have M. perstans infection. Hence, at least 44 cases and 132 controls should be enrolled in the study, with a 1:3 matching ratio.”*

Detailed information about the matching can be found in the methodology, results and discussion.

We now added in the methods:

*“The study was designed to be a case control study where cases and controls were matched for age and village.”*

And in statistical analysis we now state:

*“The controls of the persons with epilepsy not meeting the NS and OAE criteria were individually matched to the closest persons with epilepsy meeting the former, respectively.”*

**Reviewer**

2. Were correct statistical analysis used to support conclusions? Yes conditional logistic regressions model have now been reported. However, table 3 that seems to have the main issues being reported on, does not quite match what are reported in the text. There is no clear labeling of table 3 with crude and adjusted OR columns. In the text, there seems to be report of both crude or univariate as well as adjusted ORs. Perhaps the table could be made more conventional and easier to read. Maybe a biostatistician would be required to review the manuscript and ensure the reported ORs are appropriate for what are being discussed.

**Response**:

To enhance the clarity of Table 3, we have made improvements by including a new label: "level of association (OR)." Since we performed a conditional logistic regression analysis that was matched for village, age, and sex, it is no longer appropriate to mention crude/adjusted odds ratios (ORs). We appreciate the reviewer's attention to detail in highlighting the importance of adjusted ORs in our results, when we explore the association between two of the explanatory variables (Ov16 seropositivity with a family history of seizures) through a logistic regression adjusted for age, sex and village type. The latter is only presented in the text, additionally to the main conditional regression. To avoid any confusion with the main results, we have moved this paragraph to the end of the results section. We have also provided further clarification in the methodology section by explicitly stating these adjustments and their significance:

*“Correlations between predictors were explored, and a logistic regression adjusted for age, sex and village type was used to explore the association between Ov16 seropositivity and family history of seizures.”*

**Reviewer**

Does the analysis presented match the analysis plan? Yes

3. Are the results clearly and completely presented? I have challenges following the reporting of the results especially where the odds ratios are being reported; mainly in table 3. I have alluded to this earlier on (see above). Note that the results in the abstract in the online system still has the odd results as per ordinary logistics regressions.

**Response**: Kindly find the response to the previous question, where we improve the readability of Table 3. We thank for the reviewer for noticing the abstract in the online system has not been updated yet and we will ensure the correct one is used.

**Reviewer**

Are the figures (Tables, Images) of sufficient quality for clarity? Yes

4. Are the conclusions supported by the data presented? For the most part, yes. But what if there were sizeable proportions of controls with evidence of M perstans but no M perstans among the cases – wat would your conclusions have looked like on the main hypothesis that OV is but not M perstans? This is not a counterfactual situation but real. Both cases and controls have 0.0% M perstans? What does that mean should controls have had M perstans and cases did not?

**Response**: As reported in South Sudan, we were expecting to find cases and controls with *M. perstans* infection if the latter was causing nodding syndrome (NS) in the area (we now describe this in the sample size calculation previously mentioned). If there would have been more *M perstans* infection in controls than in cases this could have suggested that a *M. perstans* infection could protect against nodding syndrome. However, our results show that *M. perstans* is not endemic in the study area. Therefore, the role of *M. perstans* as a risk or protective factor in the study area could not be assessed.

**Reviewer**

5. Are the limitations of analysis clearly described? There maybe need to explicitly report that the study only two measured putative risk for epilepsy and nodding syndrome. Whatever were not measured in the cases and controls may not have balanced out just by virtue of matching on 3 variables. Issues of trauma, and other risk factors out forward by other scholars could not be assessed and this ought to come out clearly as a limitation before the string conclusion that OV is a risk factor. What if something else other than M Perstans lurks behind OV, and that something else not measured to be adjusted for?

**Response**: While it is possible that there may be other risk factors for epilepsy and nodding syndrome that were not assessed in our study, we would like to highlight that 106 individuals (94%) with epilepsy in our study met the criteria of the OAE case definition. This case definition includes the exclusion of other obvious causes of epilepsy, such as pre-natal complications, head trauma, severe malaria, measles, and meningitis.We also cannot exclude that some epilepsy was caused by neurocysticercosis. However, the latter tends to happen in adult life (which is an exclusion criterion for OAE). Moreover, in Mahenge, pigs are often kept in enclosed spaces and in suburban villages, which incidentally have significantly lower prevalence of epilepsy (see reference below).

Reference: Bhwana D, Mmbando BP, Dekker MC, Mnacho M, Kakorozya A, Matuja W, et al. Clinical presentation of epilepsy in six villages in an onchocerciasis endemic area in Mahenge, Tanzania. Epileptic Disord. 2019;21(5):425-35.

**Reviewer**

6. Do the authors discuss how these data can be helpful to advance our understanding of the topic under study? Very much so. Is public health relevance addressed? Yes but some about the use of treated bed nets and stigma seem quite extant.

**Response**: Indeed, we provide a detail discussion of the treated bed nets and stigma. Stigma is a substantial burden for persons with epilepsy. As mentioned, stigma can lead to social isolation, including situations where family members may avoid sharing meals, rooms, or beds with individuals with epilepsy. This social behaviour can have implications not only for the epidemiology of epilepsy but also for other diseases. Thank you for your valuable input and for highlighting these important points.

**Reviewer** **3**

**Reviewer**

We thank the authors for taking into account the comments of the different authors, which has greatly improved the quality of the paper and thus its relevance. The clarification of the methodological aspects brings a better understanding of what has been done. However, some questions, suggestions and comments remain.

Comment 1

Regarding the association between epilepsy and a family history of epilepsy, it is good that you have mentioned the role of genetics in your discussion. However, your discussion does not explain (or hypothesise) why the association between epilepsy in general is "much" stronger with a second-degree relative than with a first-degree relative. Indeed, whether one considers the geographical proximity of the families or the genetic aspect, one would expect the association to be stronger with first-degree relatives. Similarly, why do you think the presence of epilepsy in in-laws is "much" stronger with NS than with other types of epilepsy? This point was not raised in your discussion either, yet it seems to me to be an important point in your results

**Response**:

Thank you for the pertinent comment. In onchocerciasis-endemic sites with relatively high ongoing transmission of *O. volvulus*, such as the situation in Mahenge prior to the implementation of bi-annual CDTI, the risk of developing epilepsy is primarily determined by two factors: exposure to infected blackflies and the age of the person. Therefore, a high prevalence of epilepsy among siblings is seen in such settings. However, the number of siblings with epilepsy are expected to decrease as soon as an effective onchocerciasis elimination intervention, such as bi-annual CDTI, is implemented.

Ultimately, high Ov16 seroprevalence is expected among groups with similar ages living in an onchocerciasis-endemic area with a high prevalence of onchocerciasis-associated epilepsy, such as Mahenge. This was indeed confirmed in our study, and we added to our results:

*“Most epilepsy cases (92.3%, 12/13) and controls (100.0%, 10/10) with a sibling with epilepsy were Ov16 seropositive (**one case and one control were not tested).”*

The similar exposure of cases and controls to infected blackflies together with the small sample size of the study, explains why we were unable to show an association between first degree relatives and epilepsy. We now state in the discussion:

Being a first-degree family member epilepsy was strongly associated with Ov16 seropositivity. This was expected as direct family members generally live in the same household and are equally exposed to the *O. volvulus* parasite. Genetic factors may indeed play a role, possibly on the immunological response to the infection. Kindly find in the discussion:

*“A person with a second-degree relative with epilepsy had higher odds of having epilepsy, including probable NS. This observation is consistent with the fact that family members, especially first-degree relatives, often live in the same neighbourhood and thus share similar exposures to O. volvulus-infected blackflies (34). This is supported by the fact that individuals who tested Ov16 seropositive were more likely to have a first-degree relative with epilepsy. Notably, almost all epilepsy cases (92.2%) and all controls with a sibling with epilepsy tested seropositive. This finding further suggests that the association between epilepsy and direct family members with the condition is likely related to onchocerciasis exposure. The lack of a significant association between epilepsy and having a first-degree relative with the condition can be attributed to the comparable exposure of cases and controls to blackfly bites and the small sample size of the study.”*

The association between epilepsy and being a second-degree relative was also to be expected. We now state in the discussion:

*In contrast, a significant association was observed between being a second-degree relative and epilepsy (p<0.001). The increased presence of uncles/aunts, cousins and nephews with epilepsy in case families can likely be attributed to their shared living environment or proximity to the cases, placing them at similar risk for developing OAE.”*

The same explanation may explain the association between epilepsy and being an in-law. However also epilepsy related stigma may play a role. We now state in the discussion:

*“A person with an in-law with epilepsy had higher odds of having OAE (OR: 4.35, 95% CI: 1.04–18.20) or NS (OR: 11.04, 95% CI: 1.39–87.43). The latter may be explained by the in-law residing in the same high-risk area for onchocerciasis as the cases and the impact of epilepsy-related stigma. The stigma surrounding epilepsy can lead to social challenges, including finding a marital partner for individuals with epilepsy and their family members (35, 36, 37). For instance, a recent study in Mahenge reported that a mere 15% of persons with epilepsy were married (38). It is, therefore, plausible to consider that individuals from families affected by epilepsy may be willing to marry a person with epilepsy. Furthermore, it has been shown that persons with epilepsy in Africa frequently marry community members of lower social status, further emphasising the influence of social dynamics in forming relationships (37).”*

In addition, we found that while Ov16 seroprevalence was high in both cases and controls, it was notably higher in cases. Hence, we added in the discussion that we anticipate that the microfilarial load will be higher among cases, as previous studies have established its association with onchocerciasis-associated epilepsy, including NS:

*“There were at least two children with epilepsy in 14 (10.8%) of the 113 case families, while in 19 (9.9%) of 132 control families, there was at least one child with epilepsy. The latter suggests that control families were likely exposed to a lesser extent and duration to infected blackfly bites than the families of cases, as evidenced by the higher Ov16 seroprevalence of cases and longer family residence in the study area (p<0.05). Hence, it is anticipated that cases would exhibit higher microfilarial loads (35).”*

**Reviewer**

Question: Have you considered the possibility that there is a gene (or genetic predisposition) that predisposes to both NS and OV infection? This would explain the epidemiological association found between NS and OV? What do you think?

**Response**: Indeed, there could be a genetic predisposition for both developing NS and an Ov infection with a higher microfilarial. A future study could look at this hypothesis. We now have in the discussion:

*“The higher epilepsy prevalence among relatives of cases may indicate that the clustering of epilepsy in families could also be due to a potential genetic predisposition, where certain families could be more susceptible to both Onchocerca parasite infection and NS development. Still, this association between epilepsy and having a family member with epilepsy was lost when the conditional regression was adjusted for Ov16 seropositivity, indicating that exposure to O. volvulus played a more significant role in the observed relationship. ”*

Our research team is currently exploring a possible link of NS with HLAs in Mahenge.

**Reviewer**

Comment 2

In Table 3, regarding the "number of years spent in the village", you do not indicate for which "year gap" the OR is calculated. I assume that it is the OR for a one year gap. However, given the medians, I wonder if a one-year difference is meaningful for comparing two people in this population. I suggest you redo the model by comparing two individuals with a one standard deviation years difference in time spent in the village.

Also in Table 3, why did you not run a multivariate conditional regression model on each epilepsy with the following covariates: Ov16, family history of epilepsy and time spent in the village? Is this also a missing data problem? If not, I think it should be done and these results would be more accurate like the association between family history of epilepsy and epilepsy that disappears when the tests (Ov16) are taken into account and that you mentioned in the discussion.

**Response**: Indeed, the variable “Years family lived in the village” is annual and continuous. A sensitivity analysis was done by categorising this variable. However, it was not feasible due to the limited study size. Although not obvious from the median and IQR of the variable, there was a difference in the years lived in the study area in the matched cases and controls, with cases usually living 1-3 years more (which, when linked to their current age and the age they developed epilepsy, it may play a major role as onchocerciasis-associated epilepsy starts in childhood years). Moreover, the variables did not follow a normal distribution (Shapiro-Wilk normality test of p < 0.001). Hence, we suggest keeping the statistical analysis as it is.

We conducted a conditional logistic regression analysis, taking into account age, sex, and village covariates. However, we did not include a multivariable conditional regression model that incorporated all three significant predictors (Ov16 seropositivity, family member with epilepsy, and number of years lived in the study area) due to their correlation. Including them together would have resulted in multicollinearity issues given our sample size. Notably, the correlation between the Ov16 variable and having a family member with epilepsy was particularly strong, as almost all cases and controls with a sibling with epilepsy tested positive for Ov16 antibodies. We have updated the results accordingly.:

*“Moreover, most epilepsy cases (92.3%, 12/13) and controls (100.0%, 10/10) with a sibling with epilepsy were Ov16 seropositive (one case and one control were not tested).”*

This correlation was expected, because the Ov16 exposure increases as a function of time lived in the study area (where blackfly biting rates are known to be higher than in the surrounding settlements) and when a sibling is seropositive, the remaining siblings are also likely to be seropositive due to similar *O. volvulus* exposures.

We thank the reviewer for the time and consideration in reviewing our paper.

**Reviewer 4**

The study objectives are clearly articulated, with appropriate study designs addressing the objectives. Study population and sample size are well described and correct statistical analysis were used. Ethical issues were well addressed.

The study results are clearly and well presented.

Conclusion and recommendation are clearly stated.

We thank the reviewer for the time and consideration in reviewing our paper.
